# Soil microbial drought history affects physiological response of select tree species to drought stress

**DOI:** 10.1007/s00442-026-05917-2

**Published:** 2026-06-26

**Authors:** Nicole M. Spanier, Richard P. Phillips

**Affiliations:** Indiana University Bloomington, Bloomington, IN USA

**Keywords:** Anisohydry, Drought sensitivity, Gas exchange, Isohydry, Legacy effects

## Abstract

Soil microbes aid in the drought tolerance of plants, yet the extent to which a microbial community’s previous drought exposure can affect plant responses to contemporary drought is largely unknown. To address this gap using a greenhouse experiment, we planted saplings of three eastern hardwoods (*Prunus virginiana*,* Liriodendron tulipifera*, and *Quercus rubra*) in field soils exposed to experimentally-induced drought or ambient moisture. Then, we altered the watering regime of the pots to induce contemporary drought (or no water stress) and measured several physiological responses. In pots containing soils with no drought history, contemporary drought reduced C assimilation rates, stomatal conductance, and leaf water potential in all tree species, as expected. However, in pots containing soils with a drought history, one species – *P. virginiana* – was buffered from the effects of contemporary drought, displaying no drought-induced changes in physiology. The buffering effect of the soil drought history treatment also affected soluble sugars, which increased to a lesser degree (in response to contemporary drought) than in soils with no drought history. These effects were not apparent in the other two species. Sterilized soils suggest that the soil drought history effect on some physiological variables in *P. virginiana* likely resulted from drought-induced responses of soil microbes as opposed to soil physical or chemical changes. Collectively, our results indicate that in addition to plant hydraulic traits, the physiological responses of some tree species to drought may depend, in part, on plant-soil-microbe interactions shaped by past stress exposures.

**Supplementary Information:**

The online version contains supplementary material available at 10.1007/s00442-026-05917-2.

## Introduction

Predicted increases in the frequency and intensity of drought (Büntgen et al. 2021; Masson-Delmotte et al. 2022) have prompted interest in quantifying the drought sensitivity of forests, one of the largest carbon (C) sinks on earth (Pan et al. 2011). Most studies of tree drought tolerance have focused on hydraulic traits of the dominant tree species (Anderegg 2015; Anderegg et al. 2016; Choat et al. 2018), with limited consideration of how soil processes (including soil microbiomes) affect tree water use. Microbial mediation of plant drought tolerance has been reported for numerous taxa (Lau and Lennon 2012; Kumar and Verma 2018), including trees (Allsup and Lankau 2019; Allsup et al. 2023), suggesting that a perspective of drought sensitivity based on plant hydraulic traits alone may be inadequate. Linking microbial mediation of drought tolerance with other factors known to affect drought sensitivity (Anderegg et al. 2018; Kannenberg et al. 2019) may yield more robust predictions about climate change impacts on the land C sink.

There are two primary ways that soil microbes affect plant drought tolerance: (1) by altering plant physiology via hormonal signaling (Hussain et al. 2014; Cohen et al. 2015) and (2) by indirectly or directly altering soil water availability (Duñabeitia et al. 2004, Pavithra and Yapa 2018). Microbes can send hormonal signals directly to plants to upregulate stomatal conductance and root growth in response to water deficits (Kumar and Verma 2018) or excrete compounds that degrade ethylene precursors, thereby releasing plants from drought-induced growth reductions (Akbari et al. 2007; Hussain et al. 2014). Additionally, certain microbes release the stress hormone abscisic acid to trigger stomatal closure, thus relieving plant water stress (Cohen et al. 2015). In terms of water availability, plant-associated microbes can increase plant access to (and uptake of) water or alter soil properties in ways that increase water retention. For instance, many plants increase associations with mycorrhizal fungi when exposed to water-stress, as the fungi can extend hyphae into small soil pores to access water (Augé et al. 2001; Duñabeitia et al. 2004, Smith et al. 2010; Pavithra and Yapa 2018) and increase water retention by binding soil particles together (Augé et al. 2001). Likewise, root-associated microbes that excrete extracellular polymeric substances (EPS), which enhance the formation of water-retentive aggregates, are often promoted under drought stress (Alami et al. 2000; Costa et al. 2018). While there is compelling evidence that microbes can affect plant drought tolerance, we lack understanding of whether such dynamics are widespread or relatively rare, especially in woody plants (Phillips et al. 2016).

Given that plant-associated microbes have been shown to alter stomatal conductance (Forchetti et al. 2007; Salomon et al. 2014; Cohen et al. 2015; Tsukanova et al. 2017), an open question is whether microbes affect plant hydraulic strategies and excess C storage. Some plants close stomata at the first signs of drought (strict isohydry), some keep their stomata open during drought and make other physiological adjustments (strict anisohydry), and some adopt a strategy that is intermediate between the two extremes (Tardieu and Simonneau 1998). If plant-associated microbes secrete hormones (e.g., abscisic acid) in response to drought to trigger stomatal closure, the plant’s hydraulic strategy may shift (Tardieu and Simonneau 1998; Kannenberg and Phillips 2017; Tsukanova et al. 2017). In addition, non-structural carbohydrates (NSCs), which are produced by plants when C supply exceeds demand (Chapin et al. 1990; Kozlowski 1992; Dietze et al. 2014), are sensitive to drought-induced changes in C assimilation (O’Brien et al. 2014; Dickman et al. 2015; Tomasella et al. 2019). If soil microbes induce plants to become more isohydric, a cascade of C consequences may follow. Yet, whether microbial effects are amplified, muted, or unaffected by the drought history of those soil microbes is unknown.

Plant species likely differ in their responsiveness to microbial signals, with possible consequences for plant drought tolerance. In a study where trees were inoculated with microbes with different water stress histories, Allsup et al. (2023) found that inoculating tree species with drought-stressed microbes can increase tree survival under contemporary drought conditions. However, the response was inconsistent among tree species. Trees that associated with arbuscular mycorrhizal fungi (AMF) benefitted from the drought-stressed inoculum (owing to greater diversity of arbuscular mycorrhizal fungi), whereas trees that associated with ectomycorrhizal fungi (EMF) were insensitive to the historical conditions of their microbes and did not experience the microbial-mediated benefits under drought (Allsup et al. 2023). Thus, if plants have interspecific differences in their responsiveness to microbial influence, interspecific differences in whole-plant sensitivity to drought may occur.

Like tree species, which differ greatly in their drought tolerance mechanisms, microbes also possess adaptations for dealing with drought, and microbial community composition is highly sensitive to water stress (Lau and Lennon 2012; Ochoa-Hueso et al. 2018; Munoz-Ucros et al. 2022; Evans et al. 2022). Certain species of microbes (e.g. Actinobacteria) tolerate drought (Bouskill et al. 2013; Wipf et al. 2021; Munoz-Ucros et al. 2022) owing to their ability produce thick cell walls (Ebrahimi-Zarandi et al. 2023) and their capacity to produce osmolytes (Bouskill et al. 2013). Thus, changes in microbial community composition due to drought legacies could affect tree sensitivity to drought if such compositional shifts impact tree fitness. Such interactions may also depend on mycorrhizal fungi. Drought stressed soils can harbor a greater diversity of AMF, which may buffer trees from the negative consequences of contemporary drought (Allsup et al. 2023). Whether such mechanisms affect drought physiology in trees that associate with other types of mycorrhizal fungi is incompletely understood.

To date, much of the research on microbial mediation of tree drought tolerance has focused on how microbes affect tree growth and survival during drought, with limited consideration of morphological, physiological, and chemical responses. Moreover, few studies have explored whether the drought history of microbes affects tree physiological responses to contemporary drought. Here we use a controlled greenhouse experiment to uncover the effects of microbially-mediated drought history on trees’ response to subsequent drought conditions. *We hypothesized that trees planted with drought-exposed soil microbes will be buffered from the effects of a contemporary drought and will exhibit changes in their degree of isohydry.* By understanding how soil drought history affects tree physiology under droughts, we can better predict how trees will fare against the more frequent and intense droughts induced by climate change.

## Materials and methods

### Site description for soil harvesting

We collected soil from two adjacent forest plots in Griffy Woods, Indiana, USA (39°11’N, 86°30’W). The site is a deciduous hardwood forest dominated primarily by *Quercus rubra* (red oak), *Acer saccharum* (sugar maple), and *Liriodendron tulipifera* (tulip poplar). One plot had been exposed to ambient levels of precipitation, and therefore the resident soil microbes experienced an ambient precipitation history; we designate this soil as the “control” soil history. The other plot has been exposed to a 55% reduction of throughfall using a 40 m x 40 m throughfall displacement experiment (TDE) design (Asbjornsen et al. 2018) for ~ 4.5 years. Hereafter we use the term “drought-stressed” to refer to the soil history in this plot, as the soil and microbes have been exposed to a reduction in water inputs. The soils at Griffy Woods are silty-loams (mesic Typic Dystrudepts and Hapludults in the Brownstown-Gilwood complex) derived from siltstone and shale.

### Experimental design

We collected soil to 10 cm depth from 10 locations at least 3 m from each other from each of the forest plots and homogenized the soils to generate two soils: a “control” and a “drought-stressed” soil. We ensured that five of the soil samples within each plot were taken from underneath an arbuscular mycorrhizal tree species and the other five to be taken from underneath an ectomycorrhizal tree species. In addition, we made sure at least one of the soil samples came from underneath a *Q. rubra* and one from underneath a *L. tulipifera*, as they are species used in the following greenhouse experiment. We also note that soil % C, soil % N, soil C: N, soil texture, and soil bulk density did not differ between the sites one year after initiation of the experiment (Table S6). In addition, these plots were directly adjacent to each other which means they have the same parent material, slope, and exposure to weather. Therefore, the physical and chemical variables of the pre-treatment “control” and “drought-stressed” soils are similar, allowing for the isolation of a ‘soil drought history’ effect. Notably, we pooled soil samples collected from across the TDE plot and also from the adjacent ambient (i.e. control) plot. While this approach does not allow us to capture fine scale heterogeneity in soil responses, our goal was to generate a large volume of soil to investigate how a droughted soil impacts tree physiology, which makes our pooling method appropriate (Cahill et al. 2017; Gundale et al. 2017, 2019).

In the greenhouse, we planted one- to two-year-old saplings of *L. tulipifera* (Cold Stream Farm; Free Soil, MI), *Q. rubra* (Vallonia State Nursery; Vallonia, IN), and *Prunus virginiana* (chokecherry; Cold Stream Farm; Free Soil, MI) in each of the two soils. *Liriodendron tulipifera* is a water-demanding tree that adopts an isohydric hydraulic strategy (Roman et al. 2015; Yi et al. 2017) and associates with AMF. *Quercus rubra* is anisohydric and associates with EMF (Roman et al. 2015; Yi et al. 2017). We selected *Q. rubra* and *L. tulipifera* given that both are commonly found at Griffy Woods. *Prunus virginiana* was not part of the original study design; however, after the local nursery accidentally sent us *P. virginiana* saplings, we decided to include it in order to increase the number of species. Though uncommon in southern Indiana, a closely related congener, *Prunus serontina* (black cherry), is a component of Griffy Woods. The hydraulic traits of *P. virginiana* are unknown, though like *P. serontina*, the species associates with AMF (Bainard et al. 2011). Given that the saplings used in this study were nursery-grown (and not sown from sterilized seeds), we cannot rule out microbial colonization of trees (e.g., by mycorrhizal fungi or endophytes) prior to our experimental inoculum addition. However, any microbial carry-over effects from the nursery would be consistent across treatments and would likely make our soil treatment effects conservative owing to well-described priority effects (Kennedy et al. 2009; Kong et al. 2025).

**Fig. 1:**
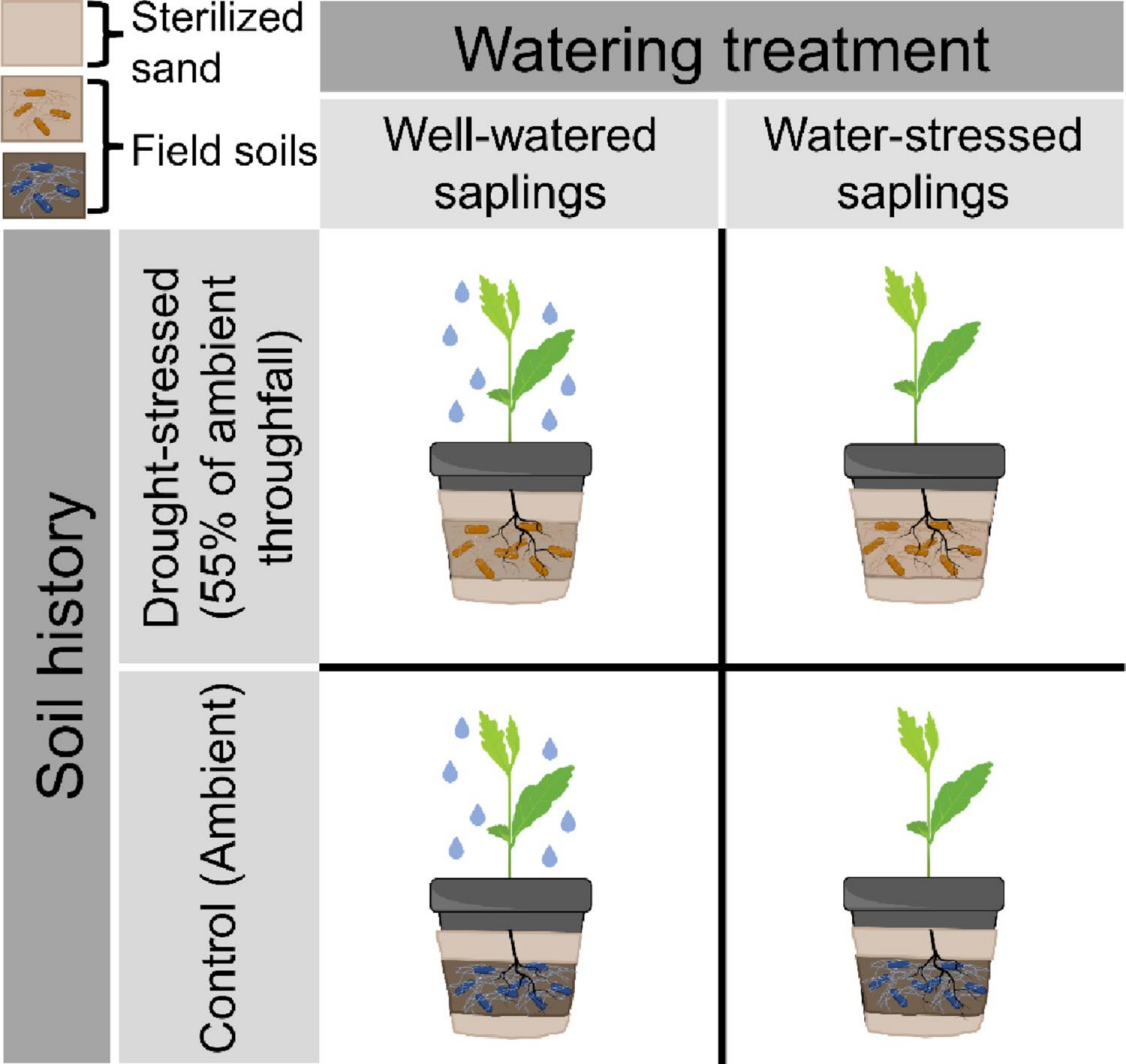
Visual representation of the experimental design. The soil color represents the drought history of that soil and the resident microbes. The water droplets represent the experimental watering treatment we imposed on the saplings

When planting, we first placed a 1.5–2 L layer of sterilized sand in a tall tree pot and then positioned the sapling in the pot. We placed approximately 5 L of the appropriate field soil to completely cover the root zone of the tree and added 1–1.5 L of sterilized sand on top of the field soil (Fig. 1). We used this sand-soil-sand layering design to minimize the amount of field soil removed from the field site and accommodate for the large size of the tree pots compared to sapling size. We acclimated the plants for 3 weeks starting in May 2022, allowing them to leaf out before starting the watering treatments and measurements. We submitted the saplings to either a well-watered (weekly watering) or water-stressed (biweekly watering or “contemporary drought”) watering treatment. For each of the treatment combinations, we had six replicate trees (*N* = 72, 24 trees per species). In addition, for each of the treatment combinations, we had three replicate trees (*N* = 36, 6 trees per species) but planted those trees in a subset of soils that was sterilized using an autoclave (1 h at 120℃).

### Physiological measurements

We measured spot leaf water potential (Ψ_Leaf_) using a Model 610 Scholander-type pressure chamber (PMS Instrument Company, Corvallis, OR) at the midpoint of the experiment in July 2022. At the conclusion of 10 weeks within +/- three hours of solar noon in September 2022, we measured photosynthetic assimilation (*A*) and stomatal conductance (*gsw*) once on one canopy leaf per tree using the LI-COR 6800 (LI-COR Inc., Lincoln, NE). We set the chamber conditions to a constant 600 µmol/s flow rate, 60% relative humidity, 420 µmol/mol CO_2_ concentration, 10,000 rpm fan speed, 29 °C temperature, and 1500 µmol/m^2^/s light setpoint. We calculated intrinsic water use efficiency (iWUE) by dividing *A* by *gsw*. For the well-watered trees, we measured Ψ_Leaf_, *A*, and *gsw* one week after watering them, and for the water-stressed trees, we took these same measurements two weeks after watering. We then determined the tree hydraulic strategy for each treatment to establish if soil history influenced tree hydraulic status. To determine hydraulic strategy, we first watered the trees to saturation after measuring *A* and *gsw*. We then measured Ψ_Leaf_ every other day for two weeks while the soil dried out to capture differences in Ψ_Leaf_ with changing soil moisture levels. We simultaneously measured soil volumetric water content (%) using a Hydrosense II meter (Campbell Scientific, Logan, UT). We then converted volumetric water content to soil water potential (Ψ_Soil_) using a soil water retention curve that was created using soil psychrometers (WP4-C, Decagon Devices Inc., Pullman, WA, United States) on our sand-soil mix upon the completion of our experiment. We saved the leaves used for Ψ_Leaf_ measurements to later determine total leaf dry biomass. On the day of *A* and *gsw* measurements, we also measured volumetric water content and converted them to Ψ_Soil_ using the same method shared above to determine level of water deficit.

### Growth and biomass

We measured stem elongation and stem diameter at the beginning of the experiment and at the end. We measured the distance from the base of one randomly selected branch per plant to the tip to track stem elongation. In addition, we used calipers to measure stem diameter growth at the base of the branch used for stem elongation. We calculated stem elongation and diameter relative growth rates (RGR) by subtracting the initial measure from the final measure then dividing by the initial measure. After the conclusion of the experiment, we harvested the trees and separated them into stems, leaves, and roots. We wet-weighed the stems and leaves, dried them at 60 °C for at least 48 h, and dry-weighed them for biomass. For root biomass, we first subsampled the roots for morphological analyses, which is detailed in the subsequent section. We wet-weighed both the larger mass of roots and the subsample. We then dried the larger root mass at 60 °C for at least 48 h and dry-weighed the sample. We determined the linear relationship between wet weight and dry weight for each of the species, and from this relationship, we calculated the inferred dry weight of the root subsample and added this to the final dry biomass measurement.

### Root morphology

Root subsamples collected after harvesting were scanned using an EPSON GT-20,000 flatbed scanner (Epson, Nagano, Japan) at 600 dpi. We took particular care to spread the roots out for more accurate structural analyses. We analyzed root morphology and architecture using the software *Rhizovision* and following the protocols described in Seethepalli and York (2020). We investigated if there was a difference in the average branching intensity (BI), diameter (D), and specific root length (SRL) among treatments.

### Nitrogen concentration and non-structural carbohydrates

For N concentration and NSC analyses, we ground the leaf samples using a SPEX 2010 GenoGrinder (SPEX^®^ Sample Prep, Metuchen, NJ, USA), and we ground the stem and root samples using a Thomas Scientific-Wiley Mini-Mill (Thomas Scientific, Swedesboro, NJ, USA). We measured two types of NSCs, soluble sugar and soluble starch, from each of the tissue types (stem, leaf, and root) once per tree using an extraction protocol adapted from Chow and Landhausser (2004). We extracted the soluble sugars in a liquid phase using a mixture of methanol, chloroform and water, while starches were precipitated in the form of a starch pellet. We depolymerized the starch pellet using diluted sulfuric acid in a 90 °C water bath for 30 min. We then took the resulting soluble starches and sugars and added concentrated sulfuric acid and 2% phenol. In a dark room, we allowed the resulting yellow color to develop for 10 min after mixing. We used a UV-1700 spectrophotometer (Shimadzu Corporation, Kyoto, Japan) to determine the colorimetric concentration of soluble sugars and starches in the solution at a wavelength of 490 nm. We converted spectrophotometric data to NSC concentrations (% dry mass of the plant tissue) using 1:1:1 D-glucose: D-fructose: D-galactose for the standard curve and the total dry mass of the tissue (Kannenberg and Phillips 2020). To determine the N concentrations, we analyzed the ground material from each tissue, once per tree, using the Elemental Combustion System 4010 (Costech Analytical Technologies, Valencia, CA, USA).

### Statistical analyses

We performed all statistical analyses and created all visualizations using R (Posit Team 2025). We first verified the data were normal with Shapiro-Wilk tests. We then ran a linear model of response~Soil history*Species*Watering treatment to investigate the effects of watering treatment, soil history, species, and their interactions using the package “car” (Fox et al. 2001) and did a pairwise comparisons with Bonferroni corrections to reduce false discovery rates using the package “emmeans” (Lenth 2017). We ran linear models of response~Soil history *Watering treatment*Sterilization for each species to investigate the effect of soil history compared to the sterilized controls using the “car” (Fox et al. 2001) and “emmeans” packages (Lenth 2017). We also computed Hedges’ g effect size and the 95% confidence intervals to reflect the difference the water-stressed treatment made on a measure within each of the soil history treatments. We selected Hedges’ g, as it has been identified as a measure of effect size that is often optimal for studies with small sample sizes (Grissom and Kim 2005). To calculate Hedges’ g, we used the package “effsize” (Torchiano 2016). To compare hydraulic strategies, we employed a linear-mixed effects model with the package “nlme” (Pinheiro et al. 2017) that investigated how leaf water potential varied with soil water potential, water treatment, microbial history, and their interactions. In addition, we accounted for temporal autocorrelation from repeated measures using an AR(1) autocorrelation function in the model.

## Results

### Impact of contemporary drought on physiological responses

While trees responded to elevated water stress by decreasing photosynthesis, conductance, and Ψ_Leaf_ (Table 1, Table S1), exposure to soils with a history of drought stress influenced one species more than others (Table S1, *P* < 0.05 for each physiological response~Soil history*Species* Watering treatment). Water-stressed saplings experienced a 192% lower Ψ_Soil_ compared to the well-watered saplings, decreasing from − 0.336 MPa to -0.981 MPa (*P* < 0.0001).

**Table 1:**
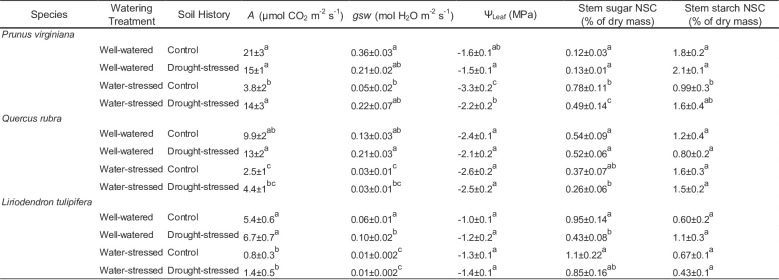
Means ± standard error of *A* (photosynthetic assimilation), *gsw* (stomatal conductance), Ψ_Leaf_ (leaf water potential), stem NSC sugar, and stem NSC starch for each of the treatment combinations and species

Superscript letters represent significant differences among treatments within a species for a given measurement from pairwise tests with Bonferroni corrections

**Fig. 2:**
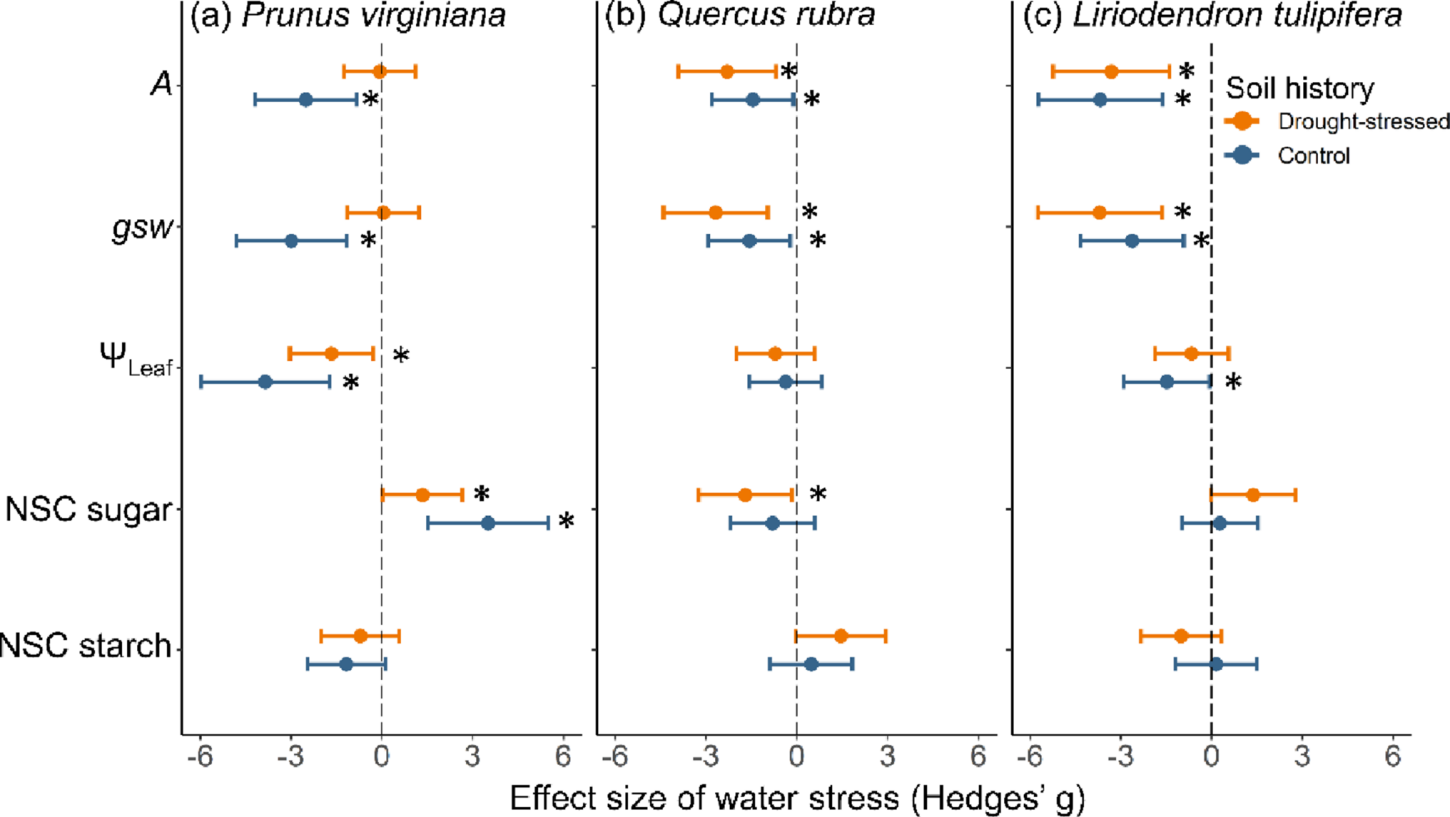
Hedges’ g effect sizes of water stress for *A* (photosynthetic assimilation), *gsw* (stomatal conductance), Ψ_Leaf_ (leaf water potential), stem NSC sugar, and stem NSC starch for (**a**) *Prunus virginiana*, (**b**) *Quercus rubra*, and (**c**) *Liriodendron tulipifera*. Effect sizes represent the difference between the well-watered saplings and the water-stressed saplings within a certain soil history that is designated by color of the points (control soil history – blue, drought-stressed soil history – orange). Error bars represent the 95% confidence intervals of the effect size, and the stars represent a significant effect of treatment

In *P. virginiana*, exposure to drought-stressed soil buffered tree responses to contemporary drought (Table 1; Fig. 2). For instance, photosynthesis was reduced by 82% in trees that experienced water stress (compared to well-watered controls) and were planted in soil with no drought history (Hedges’ g = -2.52, CI= [-4.20, -0.846], Fig. 2; Table 1), while photosynthetic rates of *P. virginiana* were unaffected by water stress when trees were planted in soils with a drought history (Hedges’ g = -0.0745, CI= [-1.26, 1.11], Fig. 2; Table 1). Importantly, sterilization had a main effect on *P. virginiana*’s photosynthetic assimilation with no interactions, suggesting that living microbes were likely responsible for the observed soil history treatment differences and indicating that microbes are also instrumental to plant physiology when under optimal moisture conditions (Table S2 and Table S3). For *Q. rubra*, both the drought-stressed (Hedges’ g = -2.30, CI= [-3.92, -0.692], Fig. 2) and control (Hedges’ g = -1.46, CI= [-2.80, -0.124], Fig. 2) soil histories did not buffer the tree’s photosynthetic assimilation under the water-stressed conditions (Table 1). The *L. tulipifera* trees planted in the soil with a history of drought-stress (Hedges’ g = -3.32, CI= [-5.25, -1.39], Fig. 2) as well as those planted in the soil with a control moisture history (Hedges’ g = -3.68, CI= [-5.74, -1.65], Fig. 2) experienced reductions in photosynthetic assimilation with further water-stress (Table 1).

The results for stomatal conductance mirrored the photosynthetic assimilation results (Table 1, Table S1, Table S3). *Prunus virginiana* experienced an 86% decline in *gsw* when water-stressed and planted with control history soils (Hedges’ g = -2.99, CI= [-4.81, -1.17], Fig. 2), but there was no effect of subsequent water-stress on *gsw* when planted with drought-stressed soils (Hedges’ g = 0.0447, CI= [-1.14,1.23], Fig. 2; Table 1). *Quercus rubra* and *L. tulipifera* experienced declines in *gsw* under water-stressed conditions when planted with control history soils (Table 1; *Q. rubra*: Hedges’ g = -1.57, CI= [-2.93,-0.212], Fig. 2; *L. tulipifera*: Hedges’ g = -2.63, CI= [-4.34, -0.925], Fig. 2,) as well as drought-stressed soils (Table 1; *Q. rubra*: Hedges’ g = -2.69, CI= [-4.41, -0.963], Fig. 2; *L. tulipifera*: Hedges’ g = -3.70, CI= [-5.76, -1.65], Fig. 2).

*Prunus virginiana* experienced only a 42% decline in Ψ_Leaf_ under water-stress when planted with drought-stressed soils (Hedges’ g = -1.67, CI= [-3.05, -0.289], Fig. 2) compared to a decrease of 110% when *P. virginiana* was planted with control history soils (Hedges’ g = -3.86, CI= [-5.97, -1.75], Fig. 2; Table 1). *Quercus rubra* did not decrease its Ψ_Leaf_ when water-stressed, regardless of soil history (drought-stressed microbial history: Hedges’ g = -0.713, CI= [-2.00, 0.578]; control soil history: Hedges’ g = -0.376, CI= [-1.57, 0.822], Fig. 2; Table 1). *Liriodendron tulipifera* only decreased its Ψ_Leaf_ under water-stress when planted in control soil histories (Hedges’ g = -1.49, CI= [-2.90, -0.075], Fig. 2), while *L. tulipifera* planted in drought-stressed soils did not change their Ψ_Leaf_ (Hedges’ g = -0.668, CI= [-1.89, 0.552], Fig. 2; Table 1).

*Liriodendron tulipifera* and *P. virginiana* saplings did not differ in their iWUE when exposed to water-stress under both soil histories and neither did *Q. rubra saplings* when planted in a control soil history (Fig. S1 and Table S4). However, when planted in soil with the drought-stressed history, *Q. rubra* water-stressed saplings had a higher iWUE compared to the well-watered saplings (Fig. S1 and Table S4), indicating a physiological response to the drought-stressed microbes.

**Fig. 3:**
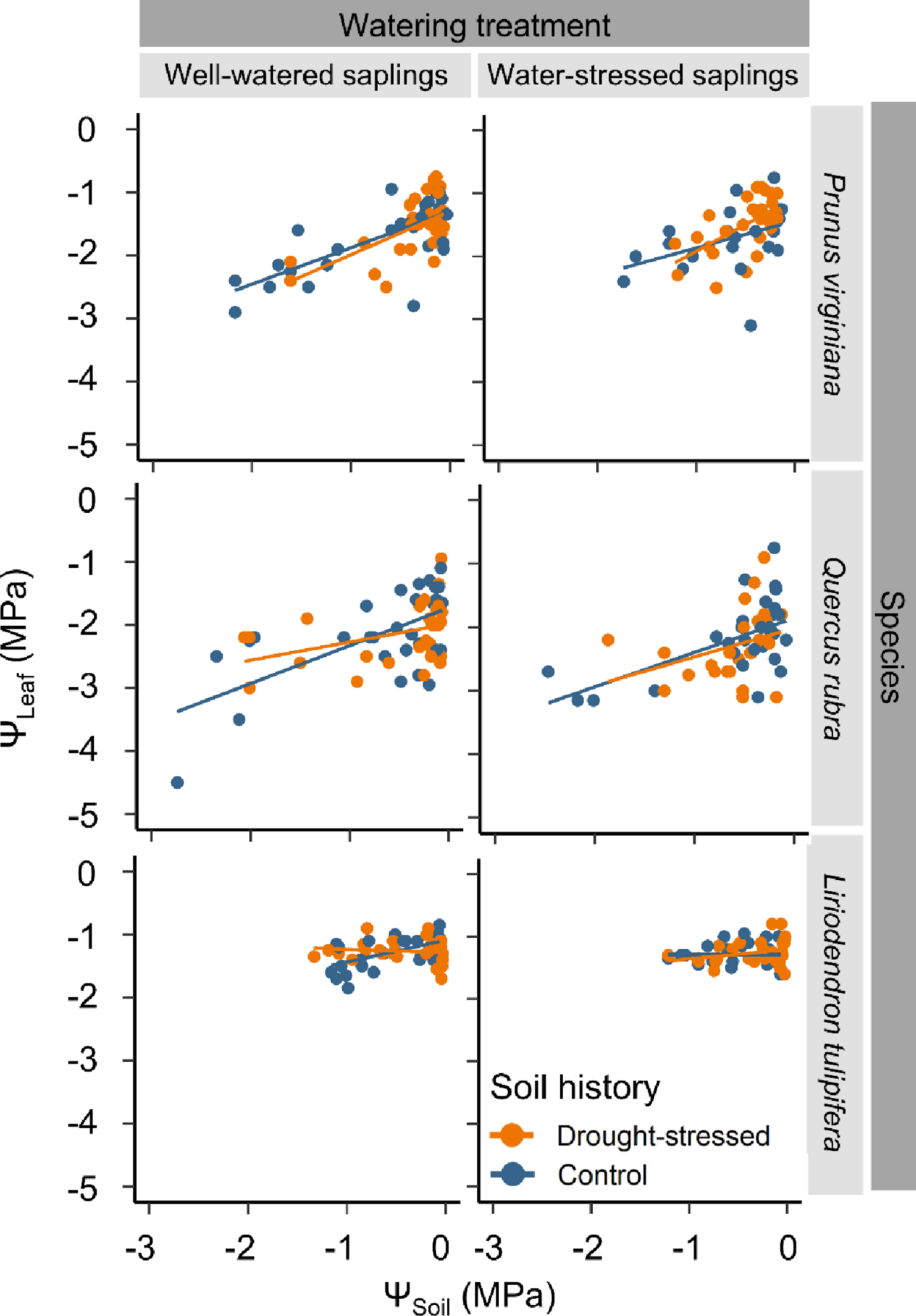
Relationships of Ψ_Soil_ (MPa) and Ψ_Leaf_ (leaf water potential; MPa) for each of the treatment combinations and species. Lines represent the line of best fit and the color of the line and data points represent the soil history (control soil history – blue, drought-stressed soil history – orange). Results of the linear mixed-effects model can be found in Table S5

Plant hydraulic strategy – the degree of isohydry versus anisohydry – was mostly unaffected by soil history. For instance, the slope of the line describing the relationship between Ψ_Soil_ and Ψ_Leaf_ was unaffected for *P. virginiana* (Fig. 3, Table S5, *p* = 0.48) and *Q. rubra* saplings (Fig. 3, Table S5, *p* = 0.43). Drought history soils pushed *L. tulipifera* trees to become more anisohydric, though only in the well-watered treatment (Fig. 3, Table S5, *p* = 0.026).

### Growth, biomass, and morphology

Watering regime and soil history had little effect on aboveground growth and root traits (Fig. S1 and Table S4). Watering treatment did not significantly alter stem elongation RGR and diameter RGR (Fig. S1 and Table S4). Root: shoot responses mirrored the patterns of RGR, as soil history did not influence root: shoot for any species (Fig. S1 and Table S4) and root: shoot in well-watered saplings were not different from water-stressed saplings (Fig. S1 and Table S4). Watering treatment did not significantly alter root BI, D, and SRL for any species, regardless of soil history (Fig. S1 and Table S4).

### Nitrogen concentration and non-structural carbohydrates

Stem N concentrations did not differ between sapling watering treatments (Fig. S1, Table S4). For *P virginiana*, root N was 27% lower in the water-stressed saplings compared to the well-watered saplings when planted in soils containing drought-stressed soils, but no differences in root N among treatments for the other species was seen (Fig. S1, Table S4). Watering treatment did not influence leaf N in *Q. rubra* saplings. However, leaf N in *P. virginiana* was 15.2% lower in the water-stressed saplings when planted in drought-stressed soil history, and in *L. tulipifera*, leaf N was 27% higher in the water-stressed saplings compared to the well-watered saplings when planted in the control soil history (Fig. S1, Table S4).

Stem NSC sugars were 562% higher in *P. virginiana* when water-stressed and planted in control history soils (Hedges’ g = 3.50, CI= [1.51, 5.49], Fig. 2), whereas those planted in soils with a history of drought increased by only 283% in response to water-stress (Hedges’ g = 1.33, CI= [0.0219, 2.65], Fig. 2; Table 1). *Quercus rubra* had 81% less stem NSC sugars under water-stressed conditions when planted in soils with a history of drought-stress (Hedges’ g =-1.71, CI= [-3.25, -0.17], Fig. 2) compared to no difference in the well-watered treatment (Hedges’ g =-0.81, CI= [-2.20, 0.59], Fig. 2; Table 1). Lastly, *L tulipifera* did not change stem concentrations of NSC sugars when water-stressed with both soil histories (Fig. 2; Table 1). There was no effect of watering treatment on stem NSC starches for all species regardless of soil history (Fig. 2; Table 1).

## Discussion

Discerning the role that soil legacies play in tree drought tolerance may lead to an improved understanding of how and why forest ecosystems differ in their sensitivity to water stress (Allsup et al. 2023). In this study, we hypothesized that previously drought-stressed soils buffer the effects of contemporary drought stress in trees, impacting their growth, gas exchange, and degree of isohydry. We found partial support for our hypothesis, as one species, *P. virginiana*, was buffered from the effects of contemporary drought when planted in soils with a history of drought-stress (Fig. 2; Table S1). This response coincided with changes in leaf soluble sugars, which increased to a lesser degree in response to contemporary drought, further indicating that the trees in drought-stressed soils experienced less overall physiological stress (Table 1; Fig. 2). Given drought history effects in *P. virginiana* were more pronounced in soils with live inoculum (Table S2 and S3), our results indicate that soil legacies - including legacies of microbial communities - can influence tree physiological responses to drought. As such, our study indicates that plant-soil interactions (and not just plant hydraulic traits) may be important yet underappreciated modulators of forest responses to drought.

The most intriguing finding from our study was that *P. virginiana* growing in soils with a drought history were buffered from changes in leaf physiology in response to contemporary drought. However, the factors responsible for the soil drought history effect are still unresolved. In general, our study supports the idea that soil microbes – and not other drought-induced changes soil characteristics – were primarily responsible for this effect. In the case of Ψ_Leaf_, soil history buffered *P. virginiana* from water stress, but only in the presence of live microbes from the soil drought history treatment (i.e., soil history*watering treatment*sterilization interaction *P* < 0.05; Table S3). For *gsw*, this buffering mechanism was also present, though evidence for this is less robust (soil history*watering treatment*sterilization: *P* = 0.055). Whether the weaker response of *gsw* (or *A*) to soil sterilization reflects a true physiological response versus a statistical power issue (owing to the limited number of replicates) is hard to say. The three-way interaction of soil history*watering treatment*sterilization was not significant for *A* (Table S3), which resulted, in part, from large standard errors in our sterilization treatment (Table S2). Notably, in the sterilization experiment, all three tree species were weakly affected by contemporary drought, even though sterilized soils had lower overall C assimilation than unsterilized soils (Table S3). This main effect of sterilization still indicates soil microbes (or microbial legacies) likely played a role in determining plant physiological responses to contemporary drought regardless of soil drought history. Thus, we interpret these findings as compelling evidence that *P. virginiana*’s mediation of drought tolerance was governed either by the activity of soil microbes or by microbially-mediated legacy effects.

One way soil microbes may have indirectly contributed to the soil history effect is via changes in microbial community composition. Although we did not sequence microbial communities in this study, it is well-established that experimental drought can alter community composition of both bacteria and fungi (Hawkes et al. 2011; Fuchslueger et al. 2014; Bouskill et al. 2016; de Vries et al. 2018). Such changes might be expected if the quantity and quality of root exudates were affected by soil drought history. Exudation rates and metabolites often change in response to drought in acquisitive, fast-growing species like *P. virginiana* (Williams and de Vries 2020). If changes in exudation stimulated certain microbes to produce more extracellular polymeric substances (EPS), Ψ_Soil_ would likely increase (Roberson and Firestone 1992; Alami et al. 2000; Costa et al. 2018). Consistent with this, *P. virginiana* pots that contained soils with a history of drought-stress had greater Ψ_Soil_ (Fig. S2) despite being irrigated with the same absolute amount of water as other pots within the water-stressed treatment. Thus, changes in EPS – resulting from exudate-induced changes in microbial activity – could have buffered the soils from drying and triggered the observed increases in *A*,* gsw*, and Ψ_Leaf_ in *P. virginiana*.

Likewise, drought-induced alteration of specific microbial guilds like mycorrhizal fungi may have contributed to the observed drought history effects. *Prunus virginiana* associate with AMF and drought-induced changes in the diversity or abundance of AMF have been linked to altered drought history effects (Allsup et al. 2023). AMF can increase plant Ψ_Leaf_ by accessing water within microsites via small diameter, high surface area hyphae (Porcel and Ruiz-Lozano 2004), thereby mitigating the effects of chronic water stress. Additionally, AMF alter plant drought tolerance by selecting for rhizosphere microbes that minimize root desiccation and enhance soil water availability (Williams and de Vries 2020). While we did not measure the mycorrhizal colonization of *P. virginiana*, the greater Ψ_Leaf_ in soils with a history of drought-stress (Table 1) indicates that *P. virginiana* had increased access to water for leaf turgor, potentially as a result of mycorrhizal-assisted water uptake or mycorrhizal enhancement of water availability (Porcel and Ruiz-Lozano 2004).

Why then did soil history have little influence on physiological metrics in *L. tulipifera*? *Liriodendron tulipifera* are strongly isohydric and close their stomata at the onset of drought (Kannenberg and Phillips 2017). As such, their soils may not dry out to the same degree, which would lessen the water stress experienced by microbes and limit microbial physiological adjustments (EPS) or activities (hyphal foraging). For instance, our biweekly watering treatment (to generate water stress) was unable to achieve especially negative soil water potentials in *L. tulipifera* pots (Fig. 3) since anticipatory stomatal closure by the trees led to reduced water uptake, altogether limiting the soil water stress experienced by the microbes and plants. This may have precluded us from detecting microbial-induced shifts in isohydric-anisohydric behavior, as reported previously (Kannenberg and Phillips 2017). Moreover, *L. tulipifera* fine roots, which are relatively thick in diameter, tend to exude less C than many heterospecifics (Yin et al. 2014). Drought has also been shown to reduce root C allocation in *L. tulipifera* in response to drought (Kannenberg and Phillips 2017). Given that exudation is believed to stimulate microbial EPS production (Redmile-Gordon et al. 2015), drought-induced reductions in *L. tulipifera* exudation rates may have limited EPS and its intending effects on soil water retention.

There was also a lack of soil history effects on *Q. rubra*’s physiology, which is more puzzling. *Q. rubra*, which are more anisohydric than *L. tulipifera* (Fig. 3), experienced drier soils which presumably would have triggered strong microbial responses such as enhanced EPS production and mycorrhizal foraging. However, *Q. rubra* also associates with EMF, which may have lesser effects on tree drought-tolerance (Allsup et al. 2023) owing to the high interspecific variability in fungal desiccation tolerance (Di Pietro et al. 2007). In addition, our results may have been conservative, as pre-established mycorrhizae (prior to our inoculation treatment) could have hindered the establishment of microbes from the drought-stressed soils. While *P. virginiana* appears to be nearly as anisohydric as *Q. rubra* (Fig. 3), rates of *A* in *P. virginiana* varied with soil water content, whereas *Q. rubra* tended to maintain its assimilation rate despite soil water content changes. Thus, the unique pairing of anisohydricity and photosynthetic sensitivity to water stress (McDowell et al. 2008) may have allowed us to detect (microbial-induced) changes in *P. virginiana* physiology but not in *Q. rubra*. Future work linking exudation rates and microbial-EPS in tree species with different hydraulic strategies should shed light on microbial mediation of tree drought tolerance.

Our stem NSC results partially align with our first hypothesis, as only *P. virginiana* NSCs indicated a physiological buffering when planted with drought-stressed soil microbes. *P. virginiana* trees increased NSC sugar concentrations under drought; however, those planted in soil with a control history had a much higher increase in NSCs than those planted in soil with a drought-stressed soil history. Since water-stress did not impact *A* and *gsw* of *P. virginiana* trees planted in drought-stressed soils, fewer osmolytes would be needed to maintain sap flow and turgor. In addition, there was an effect of water stress on *Q. rubra* NSC sugars in the drought-stressed soil history, most likely due to the slightly lower *A* and *gsw* under water stress, necessitating use of sugars for metabolism. On the other hand, there was no effect of water-stress on NSC concentrations in *L. tulipifera* regardless of soil history, an indication that its isohydric strategy led to physiological shut-down of the trees regardless of soil history. Non-structural starches were unchanged in all species, indicating that the water-stress treatment was not severe enough to trigger NSC sugar to starch conversion. These results mostly agree with Kannenberg and Phillips (2020) which found that NSC pools did not decrease for anisohydric or isohydric species, suggesting that hydraulic strategy may not be a robust predictor of NSC fluctuations (Kannenberg and Phillips 2020).

In our study, microbial effects on *P. virginiana*’s photosynthetic assimilation rates and NSCs did not coincide with aboveground growth, total biomass, or root morphology. In this way our growth results were inconsistent with Allsup and Lankau (2019), who found higher total seedling biomass under drought when seedlings were planted with microbes sourced from drier sites. Differences in the tree growth stage and the drought exposure of the inoculum may explain this paradox. The Allsup and Lankau (2019) study utilized young seedlings that were raised from seed as opposed to the one- to two-year-old saplings obtained from a commercial nursery (as in this study). Seedlings are often more sensitive to experimental drought than saplings (Cavender-Bares and Bazzaz 2000), and our short-term sapling experiment may have precluded us from detecting effects on growth due to their lower drought-sensitivity. In a similar study to Allsup and Lankau (2019), Allsup et al. (2023) found increased survivorship of seedlings under drought when planted in soils sourced from more arid sites. While we used soil with microbes sourced from a 4.5 year drought experiment, Allsup and Lankau (2019) and Allsup et al. (2023) inoculated their trees from a natural precipitation gradient where microbial drought-tolerance may have evolved over the long-term. Whether experimentally-induced droughts affect microbial communities to the same degree as moisture changes across a precipitation gradient is not well-known, though recent work indicates microbial resistance to (and recovery from) experimentally-imposed drought may be independent from a microbial community’s precipitation history (Leizeaga et al. 2021). Likewise, gas exchange responses to drought can be asymmetric to growth responses (Kannenberg et al. 2022), indicating that how trees experience drought may depend on the scale of inference. Nevertheless, whether soil history is affecting gas exchange (as shown in this study) or growth and survival (as shown in the studies of Allsup and colleagues), there is emerging evidence that plant-microbe interactions and drought legacies have the potential to shape ecosystem sensitivity to drought.

A caveat with our experimental design is that soils were collected from a single location – the site of a large-scale (40 m x 40 m), long-term throughfall displacement experiment. TDEs are expensive to deploy and challenging to maintain (Asbjornsen et al. 2018), which meant that we were unable to avoid pseudoreplication during soil collection. As such, our ability to draw inferences beyond our study population may be limited. Additionally, we cannot rule out that other soil factors – in addition to microbial drought history – may have changed in response to the drought treatments. However, on the latter point, we found no significant differences in soil physical (texture, bulk density) or chemical properties (% C, % N, and C: N) between soils from the TDE and the adjacent control plot (Table S6). While we did not measure examine microbial composition pre- and post-treatment or between control plots and the TDE plots, numerous studies that have reported shifts in microbial communities in response to drought without concurrent changes in soil chemistry or physical properties (Fuchslueger et al. 2014; Bouskill et al. 2016; Hawkes et al. 2017; de Vries et al. 2018). Thus, it stands to reason that main factor driving plant responses to droughted soils was biological.

Our study is one of the first to show that soil history influences physiological drought responses of trees differently based on species identity, which builds on the buffered growth and survivorship findings in Allsup et al. (2023). Studies solely considering how plant traits determine tree drought tolerance may therefore be missing a biotic factor: soil microbes and the histories that shape them. Without microbial mediation of drought tolerance, there could be large implications for the function and carbon storage of certain species of trees as evidenced by this experiment’s results. Overall, this study highlights that microbes that are experiencing current climate change-induced reductions in precipitation may buffer select species’ physiological stresses to future droughts, which can help us predict how ecosystems will fare in the face of climatic selective pressures.

## Supplementary Information

Below is the link to the electronic supplementary material.

Supplementary Material 1

## Data Availability

The datasets used and/or analyzed during the current study are available from the corresponding author on reasonable request.

## Code Availability

Code available on request from the authors.
