# Supplementary material for: Soil microbial drought history affects physiological response of select tree species to drought stress
Source: Oecologia. 2026 Jun 26;208(7):88. doi: 10.1007/s00442-026-05917-2 (PMC13309384; doi:10.1007/s00442-026-05917-2)
Supplement: Supplementary file 1 — Supplementary Material 1 [file 442_2026_5917_MOESM1_ESM.pdf]

Soil microbial drought history affects physiological response of select tree species to drought stress

Nicole M. Spanier[1*], Richard P. Phillips[1]

Affiliation:

(1) Indiana University Bloomington, Bloomington, Indiana

*E-Mail for correspondence: nspanier@iu.edu Phone: 812-856-1563

Supplementary figures and tables

Table S1 Results of linear model to investigate the effects of watering treatments, microbial history, species, and their interactions (response~Soil history*Species*Watering treatment). The sum of squares, df, F-value, and *P* are listed for each model of *A* (photosynthetic assimilation), *gsw* (stomatal conductance), Ψ_{Leaf} (leaf water potential), stem sugar NSC (% of dry mass), and stem starch NSC (% of dry mass). Bolding of *P*-values represents that $P < 0.05$

	df	F-value	*P*
A **(μmol CO_2 m^{-2} s^{-1})**			
[Intercept]	1	240	**<2.2e-16**
Soil history	1	3.39	0.071
Species	2	29.5	**1.8E-09**
Watering treatment	1	47.1	**5.8E-09**
Soil history*Species	2	0.241	0.79
Soil history*Watering treatment	1	5.25	**0.026**
Species*Watering treatment	2	1.25	0.30
Soil history*Species*Watering treatment	2	7.75	**0.0011**
Residuals	56		
gsw **(mol H_2O m^{-2} s^{-1})**			
[Intercept]	1	178	**<2.2e-16**
Soil history	1	2.16	0.15
Species	2	29.7	**1.6E-09**
Watering treatment	1	46.7	**6.6E-09**
Soil history*Species	2	0.331	0.72
Soil history*Watering treatment	1	3.52	0.066
Species*Watering treatment	2	1.95	0.15
Soil history*Species*Watering treatment	2	12.5	**3.3E-05**
Residuals	56		
**Ψ_{Leaf} (MPa)**			
[Intercept]	1	1980	**<2.2e-16**
Soil history	1	7.40	**0.0086**
Species	2	67.6	**9.1E-16**
Watering treatment	1	42.0	**2.3E-08**
Soil history*Species	2	5.26	**0.0080**
Soil history*Watering treatment	1	2.73	0.10
Species*Watering treatment	2	12.9	**2.4E-05**
Soil history*Species*Watering treatment	2	5.29	**0.0078**
Residuals	56		
**Stem sugar NSC (% of dry mass)**			
[Intercept]	1	285	**<2.2e-16**
Soil history	1	9.03	**0.0040**
Species	2	19.9	**3.6E-07**
Watering treatment	1	8.76	**0.0046**
Soil history*Species	2	2.04	0.14
Soil history*Watering treatment	1	0.0642	0.80
Species*Watering treatment	2	10.6	**0.00013**
Soil history*Species*Watering treatment	2	1.93	0.16
Residuals	56		
**Stem starch NSC (% of dry mass)**			
[Intercept]	1	258	**<2.2e-16**
Soil history	1	0.480	0.49
Species	2	14.1	**1.3E-05**
Watering treatment	1	0.836	0.36
Soil history*Species	2	2.41	0.10
Soil history*Watering treatment	1	0.0291	0.87
Species*Watering treatment	2	5.41	**0.0073**
Soil history*Species*Watering treatment	2	1.18	0.31
Residuals	56		

Table S2 Means ± standard error of *A* (photosynthetic assimilation), *gsw* (stomatal conductance), and Ψ_{Leaf} (leaf water potential) when planted in sterilized soil for each of the treatment combinations and species. Superscript letters represent significant differences among treatments within a species for a given measurement. NA listed for SE is a result of a sample size of 1 due to mortality of trees within that treatment group

Species	Watering Treatment	Soil History	*A* (μmol CO_2 m^{-2} s^{-1})	*gsw* (mol H_2O m^{-2} s^{-1})	Ψ_{Leaf} (MPa)
Prunus virginiana					
	Well-watered	Control	$15±6^a$	$0.08±0.10^a$	$-1.8±0.2^a$
	Well-watered	Drought-stressed	$4.8±3^a$	$0.08±0.03^a$	$-1.5±0.2^a$
	Water-stressed	Control	$6.3±2^a$	$0.06±0.02^a$	$-2.3±0.3^a$
	Water-stressed	Drought-stressed	$6.2±2^a$	$0.08±0.03^a$	$-2.5±0.2^a$
Quercus rubra					
	Well-watered	Control	$12±4^a$	$0.20±0.08^a$	$-1.6±0.3^a$
	Well-watered	Drought-stressed	$9.3±2^a$	$0.13±0.02^a$	$-2.3±0.08^a$
	Water-stressed	Control	$0.4±1^a$	$0.01±0.01^a$	$-2.6±0.2^a$
	Water-stressed	Drought-stressed	$9.6±4^a$	$0.15±0.08^a$	$-2.3±0.1^a$
Liriodendron tulipifera					
	Well-watered	Control	$6.1±0.2^a$	$0.10±0.01^a$	$-1.0±0.1^a$
	Well-watered	Drought-stressed	$5.6±2^a$	$0.15±0.07^a$	$-1.1±0.06^a$
	Water-stressed	Control	$5.5±NA^a$	$0.09±NA^a$	$-0.8±NA^a$
	Water-stressed	Drought-stressed	$1.8±0.8^a$	$0.01±0.003^a$	$-1.5±0.2^a$

Table S3 Results of linear model to investigate the effects of watering treatments, soil history, and their interactions (physiological response~Soil history*Watering treatment*Sterilization) for *P. virginiana*. The sum of squares, df, F-value, and *P* are listed for each model of *A* (photosynthetic assimilation), *gsw* (stomatal conductance), and Ψ_{Leaf} (leaf water potential). Bolding of *P*-values represents that $P < 0.05$

	df	F-value	*P*
***A* (μmol CO_2 m^{-2} s^{-1})**			
[Intercept]	1	101	**1.2E-10**
Soil history	1	0.486	0.49
Watering treatment	1	8.45	**0.0072**
Sterilization	1	6.13	**0.020**
Soil history*Watering treatment	1	9.78	**0.0042**
Soil history*Sterilization	1	3.09	0.090
Watering treatment*Sterilization	1	1.37	0.25
Soil history*Watering treatment*Sterilization	1	0.527	0.47
Residuals	27		
***gsw* (mol H_2O m^{-2} s^{-1})**			
[Intercept]	1	60.2	**2.4E-08**
Soil history	1	0.0849	0.77
Watering treatment	1	4.95	**3.5E-02**
Sterilization	1	12.3	**1.6E-03**
Soil history*Watering treatment	1	4.93	**0.035**
Soil history*Sterilization	1	0.00240	0.96
Watering treatment*Sterilization	1	3.40	0.076
Soil history*Watering treatment*Sterilization	1	4.01	0.055
Residuals	27		
**Ψ_{Leaf} (MPa)**			
[Intercept]	1	924	**<2.2E-16**
Soil history	1	4.03	0.055
Watering treatment	1	48.6	**1.7E-07**
Sterilization	1	0.475	0.50
Soil history*Watering treatment	1	1.39	0.25
Soil history*Sterilization	1	4.80	**0.037**
Watering treatment*Sterilization	1	2.69	0.11
Soil history*Watering treatment*Sterilization	1	7.35	**0.012**
Residuals	27		

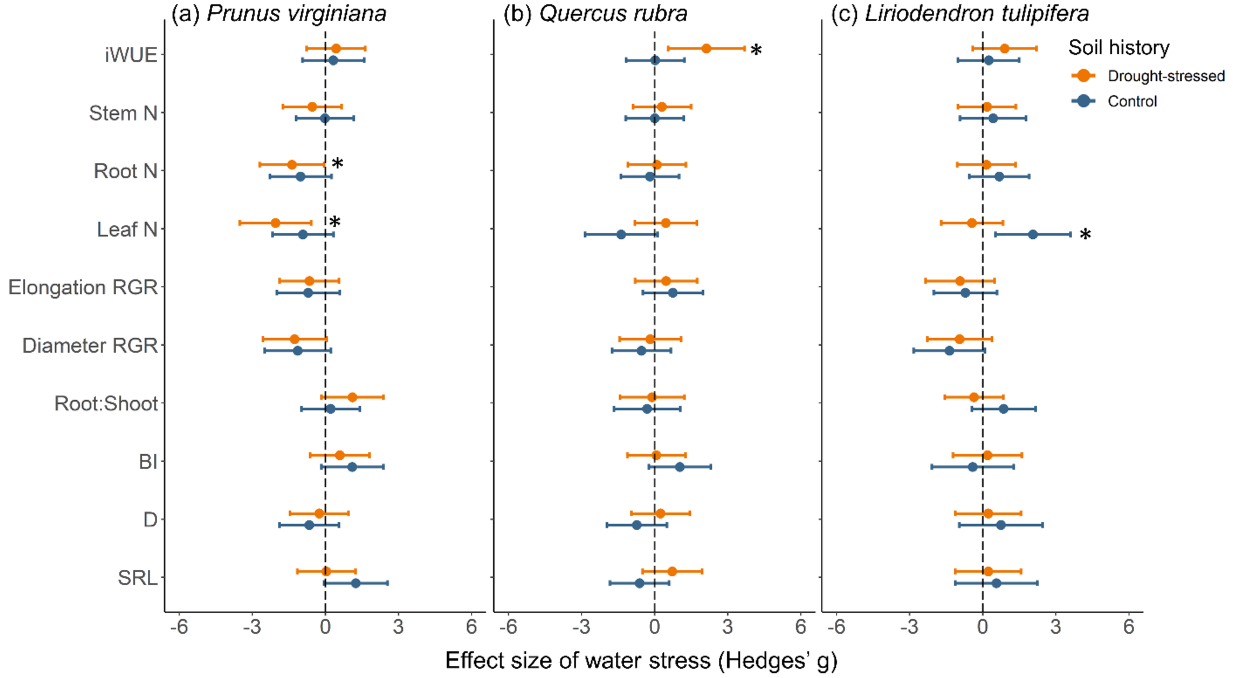

Fig. S1 Hedges' g effect sizes of water stress for iWUE (intrinsic water use efficiency), stem N, root N, leaf N, elongation RGR (relative growth rate), diameter RGR, root:shoot, BI (branching intensity), D (diameter), and SRL (specific root length) for *P. virginiana* (panel A), *Q. rubra* (panel B), and *L. tulipifera* (panel C). Effect sizes represent the difference between the well-watered and the water-stressed watering treatments within a certain soil history is designated by color of the points (control soil history – blue, drought-stressed soil history – orange). Error bars represent the 95% confidence intervals of the effect size, and the stars represent a significant effect of treatment

Table S4 Means ± standard error of iWUE (intrinsic water use efficiency), stem N, root N, leaf N, elongation RGR (relative growth rate), diameter RGR, root:shoot, BI (branching intensity), D (diameter), and SRL (specific root length) for each of the treatment combinations and species. Letters represent significant differences in variables among treatments within species

Species	Watering Treatment	Soil History	iWUE	Stem N (%)	Root N (%)	Leaf N (%)	Stem elongation RGR (cm)	Stem diameter RGR (cm)	Root:Shoot	BI (branch mm^{-1})	RD (mm)	SRL
Prunus virginiana												
	Well-watered	Control	58±3^{a}	1.1±0.11^{ab}	1.5±0.1^{ab}	2.5±0.17^{ab}	0.61±0.28^{a}	0.61±0.07^{a}	1.2±0.11^{a}	0.32±0.02^{a}	0.51±0.05^{a}	19±3^{a}
	Well-watered	Drought-stressed	70±5^{a}	1.0±0.10^{a}	1.9±0.16^{a}	2.7±0.09^{a}	0.89±0.40^{a}	0.62±0.11^{a}	1.1±0.13^{a}	0.32±0.01^{a}	0.44±0.05^{a}	22±5^{a}
	Water-stressed	Control	64±12^{a}	1.0±0.11^{b}	1.3±0.09^{b}	2.1±0.12^{b}	0.20±0.08^{a}	0.44±0.04^{ab}	1.2±0.06^{a}	0.38±0.02^{a}	0.43±0.04^{a}	24±3^{a}
	Water-stressed	Drought-stressed	79±9^{a}	0.9±0.06^{b}	1.4±0.12^{b}	2.1±0.13^{b}	0.31±0.25^{a}	0.28±0.09^{b}	1.4±0.08^{a}	0.34±0.02^{a}	0.41±0.03^{a}	22±4^{a}
Quercus rubra												
	Well-watered	Control	83±8^{ab}	0.6±0.03^{a}	1.2±0.13^{a}	2.4±0.08^{a}	0.02±0.01^{a}	0.63±0.08^{a}	1.5±0.15^{a}	0.60±0.07^{a}	0.57±0.11^{a}	10±2^{a}
	Well-watered	Drought-stressed	65±6^{a}	0.6±0.04^{a}	1.3±0.15^{a}	2.3±0.12^{a}	0.11±0.05^{a}	0.46±0.11^{a}	1.7±0.16^{a}	0.60±0.13^{a}	0.57±0.05^{a}	6.6±0.7^{a}
	Water-stressed	Control	84±19^{ab}	0.6±0.03^{a}	1.1±0.07^{a}	2.1±0.11^{a}	0.30±0.20^{a}	0.44±0.17^{a}	1.3±0.18^{a}	0.80±0.08^{a}	0.42±0.03^{a}	7.6±2^{a}
	Water-stressed	Drought-stressed	120±14^{b}	0.7±0.09^{a}	1.3±0.12^{a}	2.4±0.13^{a}	0.18±0.06^{a}	0.41±0.13^{a}	1.6±0.07^{a}	0.62±0.10^{a}	0.65±0.16^{a}	9.0±2^{a}
Liriodendron tulipifera												
	Well-watered	Control	95±11^{a}	0.7±0.12^{a}	0.95±0.06^{a}	1.6±0.08^{a}	3.0±0.82^{a}	0.45±0.07^{a}	0.73±0.07^{a}	0.65±0.28^{a}	0.39±0.02^{a}	1.8±1^{a}
	Well-watered	Drought-stressed	69±5^{a}	0.8±0.13^{a}	1.1±0.01^{a}	2.4±0.43^{a}	4.2±1.5^{a}	0.47±0.18^{a}	0.91±0.09^{a}	0.45±0.21^{a}	0.42±0.13^{a}	3.5±1^{a}
	Water-stressed	Control	112±41^{a}	0.9±0.36^{a}	1.2±0.15^{a}	2.1±0.10^{a}	1.7±0.51^{a}	0.20±0.08^{a}	0.90±0.08^{a}	0.40±0.26^{a}	0.80±0.42^{a}	7.8±4^{a}
	Water-stressed	Drought-stressed	155±47^{a}	0.1±0.45^{a}	1.1±0.10^{a}	2.0±0.14^{a}	1.9±0.67^{a}	0.19±0.03^{a}	0.82±0.09^{a}	0.53±0.04^{a}	0.48±0.07^{a}	6.0±2^{a}

Table S5 Number of degrees of freedom (df), denominator degrees of freedom, F-values, and *P*-values of the linear mixed-effects model comparing the effect of watering treatment and soil histories on the Ψ_{Soil} - Ψ_{Leaf} relationship of each species. Bolding of *P*-values represents a $P < 0.05$

	numDF	denDF	F-value	*P*
P. virginiana				
[Intercept]	1	87	1040	**<0.0001**
Ψ_{soil}	1	87	62.3	**<0.0001**
Watering treatment	1	19	0.281	0.60
Soil history	1	19	0.267	0.61
Ψ_{soil}:Watering treatment	1	87	0.00220	0.96
Ψ_{soil}:Soil history	1	87	0.239	0.51
Watering treatment:Soil history	1	19	0.886	0.36
Ψ_{soil}:Watering treatment:Soil history	1	87	0.650	0.42
Q. rubra	1			
[Intercept]	1	88	1450	**<0.0001**
Ψ_{soil}	1	88	19.0	**<0.0001**
Watering treatment	1	19	0.556	0.46
Soil history	1	19	0.700	0.41
Ψ_{soil}:Watering treatment	1	88	0.453	0.50
Ψ_{soil}:Soil history	1	88	0.466	0.50
Watering treatment:Soil history	1	19	0.0758	0.79
Ψ_{soil}:Watering treatment:Soil history	1	88	0.119	0.73
L. tulipifera	1			
[Intercept]	1	87	1950	**<0.0001**
Ψ_{soil}	1	87	4.11	**0.046**
Watering treatment	1	19	0.0373	0.85
Soil history	1	19	0.00200	0.96
Ψ_{soil}:Watering treatment	1	87	0.879	0.35
Ψ_{soil}:Soil history	1	87	0.994	0.32
Watering treatment:Soil history	1	19	0.162	0.69
Ψ_{soil}:Watering treatment:Soil history	1	87	6.18	0.015

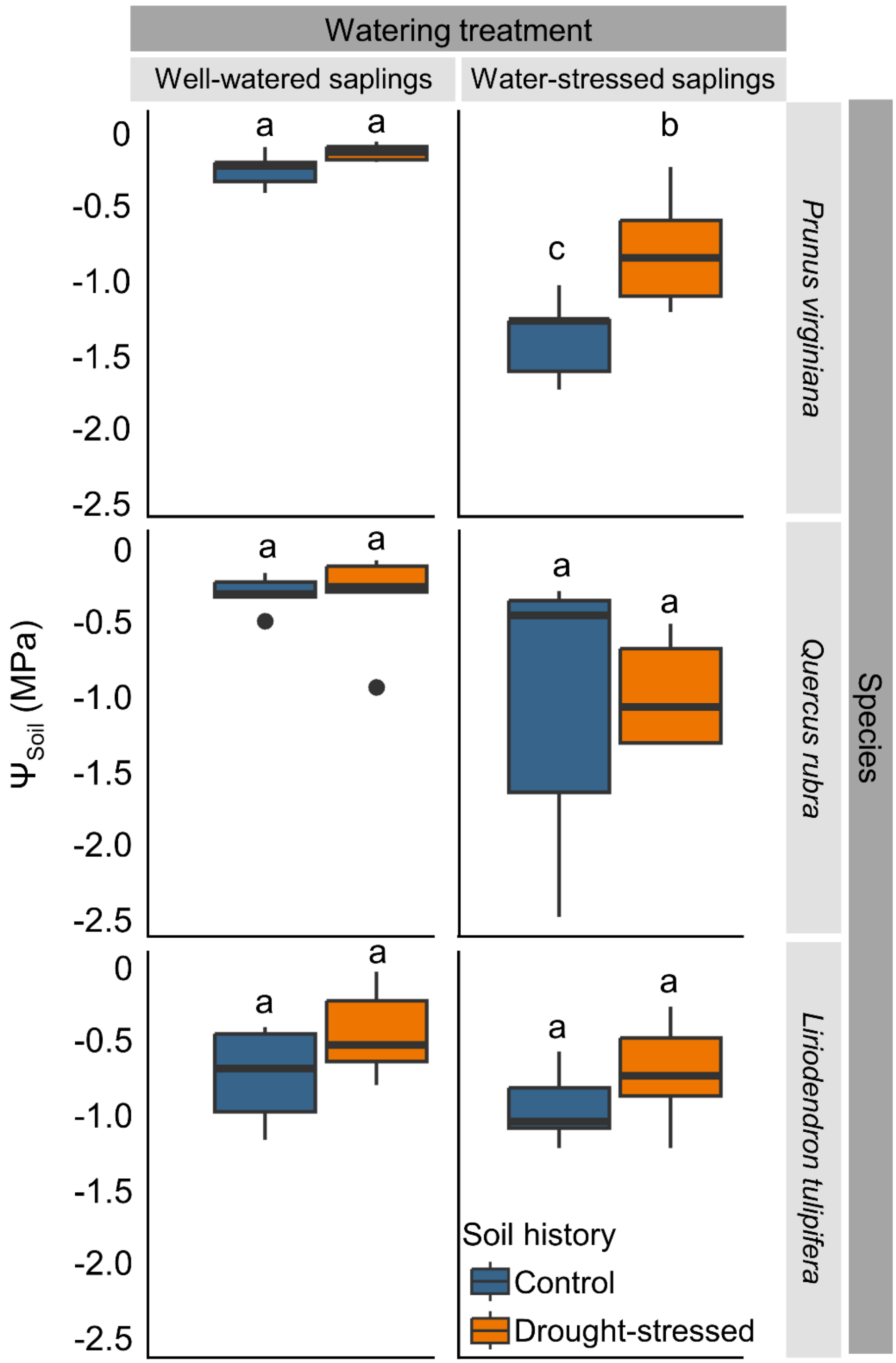

Fig S2 Soil water potential (MPa) for each of the treatment combinations and species. (control soil history – blue, drought-stressed soil history – orange). Letters above the boxplots represent a significant difference among treatment combinations for each species

Table S6 Means ± standard error of %C, %N, C:N, % sand, % silt, % clay, and bulk density (g cm^{-3}) of the soil pre-treatment from the throughfall displacement experiment (source of the drought-stressed soils) and the adjacent control plot (source of the control soils). The resultant *P*-values of a t-test between the throughfall displacement experiment and control plot values are listed for each soil variable

	**Throughfall displacement experiment**	**Control plot**	***p***
Soil % C	1.9±0.49	1.52±0.24	0.52
Soil % N	0.12±0.014	0.14±0.016	0.28
Soil C:N	27±16	11±0.37	0.33
Soil texture			
% sand	2.8±0.59	4.3±1.5	0.31
% silt	74±2.2	71±4.7	0.48
% clay	23±2.7	24.7±4.2	0.69
Soil bulk density (g cm^{-3})	1.20±0.22	1.17±0.18	0.77
